# The Secretion of Areolar (Montgomery's) Glands from Lactating Women Elicits Selective, Unconditional Responses in Neonates

**DOI:** 10.1371/journal.pone.0007579

**Published:** 2009-10-23

**Authors:** Sébastien Doucet, Robert Soussignan, Paul Sagot, Benoist Schaal

**Affiliations:** 1 Developmental Ethology and Cognitive Psychology Group, Centre Européen des Sciences du Goût (Unité Mixte de Recherche 5170), Centre National de la Recherche Scientifique - Université de Bourgogne, Dijon, France; 2 Dijon-Dresden European Laboratory for Taste and Smell (Centre National de la Recherche Scientifique, European Associated Laboratories 549), Dijon, France and Dresden, Germany; 3 Institut Fédératif de Recherche 62, Dijon, France; 4 Pôle de Gynécologie-Obstétrique, Médecine Fœtale et Stérilité Conjugale, Centre Hospitalier Universitaire du Bocage and Université de Bourgogne, Dijon, France; University of Rennes 1, France

## Abstract

**Background:**

The communicative meaning of human areolae for newborn infants was examined here in directly exposing 3-day old neonates to the secretion from the areolar glands of Montgomery donated by non related, non familiar lactating women.

**Methodology/Principal Findings:**

The effect of the areolar stimulus on the infants' behavior and autonomic nervous system was compared to that of seven reference stimuli originating either from human or non human mammalian sources, or from an arbitrarily-chosen artificial odorant. The odor of the native areolar secretion intensified more than all other stimuli the infants' inspiratory activity and appetitive oral responses. These responses appeared to develop independently from direct experience with the breast or milk.

**Conclusion/Significance:**

Areolar secretions from lactating women are especially salient to human newborns. Volatile compounds carried in these substrates are thus in a position to play a key role in establishing behavioral and physiological processes pertaining to milk transfer and production, and, hence, to survival and to the early engagement of attachment and bonding.

## Introduction

Nipples and adjacent skin (*areolae* in primates) bear a pivotal role in mammalian reproduction: they constitute the minimal areas of the females' body to enter in obligatory and recurrent contact with the offspring during lactation. Accordingly, their structure and function should be evolutionarily shaped to optimize, on the one hand, an efficient mother-to-infant transfer of water, nutrients, and immuno-protective factors carried in milk, and, on the other hand, the infant's rapid learning of sensory cues related to maternal identity and to significant events maximizing individual fitness. Indeed, in human females, the nipple-areolar region concentrates several features of potential chemo-communicative meaning directed to the suckling infant. In particular, a range of odorous substrates are locally emitted in colostrum or milk, or in the secretions of areolar glands.

Three decades of research have demonstrated that naturally-emitted volatile compounds from the breast of lactating women impinge on the behavior of human newborns in several ways. Breast odor reduces arousal states in active newborns [1], [2] and increases them in sleepy ones [2]–[4]. Furthermore, it elicits positive head turning [1], [5], [6], stimulates oral appetitive activity [3], [4], and may induce directional crawling in newborns [7].

The most studied sources of natural volatiles emanating from the breast are obviously colostrum and milk. These appear to carry arousing and attractive properties for newborns [8], [9]. Interestingly, however, the early positive bias of human newborns in favor of odor cues in human milk does not depend on prior breastfeeding experience, because neither term-born infants exclusively fed formula [9], neither premature infants [10], [11], react to these cues in the same way as do exclusively breast-fed infants. In addition, this primal attractive potency of human milk odor to newborns is not easily reassigned by engaging them to learn an artificial odorant in association with nursing [12].

**Figure 1:**
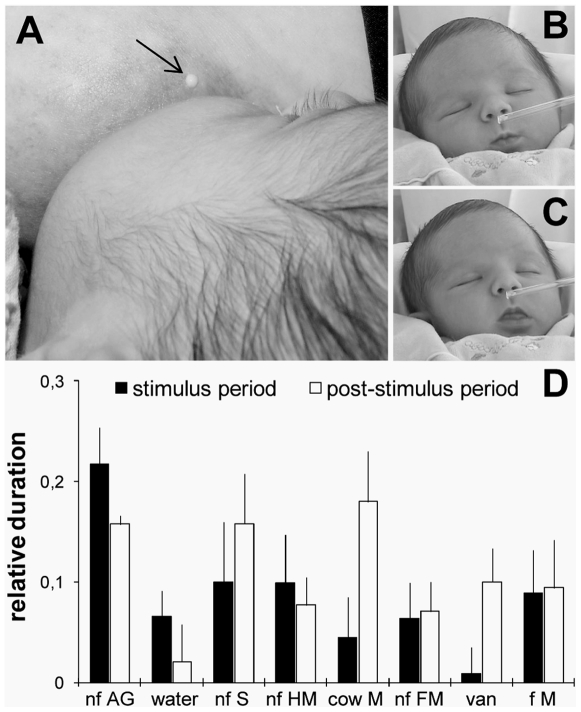
Areolar glands and infant behavior. A) Areola of a lactating woman (day 3 postpartum) with Montgomery's glands giving off their secretion (arrow). B and C) Newborns' oro-cephalic responses to the secretion of Montgomery's areolar gland (B: lip pursing; C: tongue protrusion). D) Mean (± sem) relative durations of newborns' oro-cephalic responses during (10-sec stimulus period) and after (10-sec post-stimulus period) presentation of various olfactory stimuli (Abbreviations: AG: secretions of areolar glands; S: sebum; HM: human milk; cow M: cow milk; FM: formula milk; van: vanillin; M: milk; f: familiar; nf: non-familiar; n = 19).

Another mammary source of potentially significant odor cues has received virtually no empirical consideration about its function, although it becomes morphologically conspicuous in lactating women: the glands of Montgomery. Distributed on the areolae, these glands are formed by coalesced sebaceous and lactiferous units [13]. These areolar structures enlarge during pregnancy and lactation, and can give off a noticeable latescent fluid after parturition (cf. Figure 1A) [14]–[16]. Recent data suggest that these areolar glands (AG) might be involved in the success of breastfeeding initiation, especially in first-time mothers [15], [16]. But so far, however, only correlational evidence is available, linking the mother's AG number with her perception of the infant's behavior while suckling, the timing of lactation onset, and neonatal weight regain after birth [15], [16].

Although the findings to date on the effects of AG number on infants are only correlational in nature, these findings led us to hypothesize that AG might facilitate adaptive outcomes in both infants and mothers. Accordingly, the goal of the present study was to directly assess whether human newborns detect the odorous properties of the secretions emitted by AG. Importantly, instead of presenting the stimuli “diluted” in the background of other maternal body odors (as in previous studies that exposed infants to the mixture of secretions from the whole breast, including milk [17]), we administered separately the native secretions from Montgomery's glands nasally to 3 day-old neonates and assessed their behavioral and autonomic nervous system reactions. That is, at the behavioral level, we scrutinized which responses were triggered by the odor of the Montgomerian secretion and the extent to which these may reflect the positive link between AG number and neonatal response to the whole breast [15], [16]. At the autonomic reactivity level, we analyzed whether active compounds carried in AG secretion elicit responses indicative of attentional processing (by measuring cardiac responsiveness) and whether they elicit odor sampling behaviors (by measuring alterations in infant respiratory responsiveness). The separate cardiac and respiratory response measures permitted us to characterize the *selectivity* of the biological activity of the Montgomerian secretion. In addition, we tested the effects of Montgomerian secretion against an arbitrary odor quality (vanilla), several odor substrates collected from lactating females (including both of its basic constituents such as human milk and sebum), and against heterospecific odorous mixtures, such as fresh cow's milk, cow milk-based formulas, to determine the *species-specificity* of responsiveness. Finally, we evaluated the *reinforcing potency* of the Montgomerian secretion and its dependence on postnatal experience by comparing the rate of response to its odor against responsiveness to the odor of an infant's familiar food.

## Results

### 1. Oro-cephalic responsiveness

The durations of neonatal head and mouth movements elicited by the AG secretion relative to the set of reference stimuli are depicted in Figure 1. The AG stimulus elicited a clear increase in the duration of oro-cephalic actions, which is revealed both by comparison with the pre-stimulus baseline level and by comparing the stimulus-dependent response with that to the blank control (Table 1). The AG secretion was the only stimulus to increase the relative duration of oro-cephalic actions clearly above 20% from the baseline. For this stimulus, 63% of the neonates achieved this response criterion. This proportion of responding neonates was significantly higher than for all reference stimuli [water: 21%; sebum: 21%; human milk: 26%; cow's milk: 26%; formula milk: 16%; vanillin: 5%; and familiar milk: 16%; Friedman's analysis of variance (ANOVA), F(19,7) = 19.13; *p<0.01*; all pair comparisons (using Fisher's χ^2^ test) between AG secretion and all other stimuli, *p<0.05*].

**Table 1:** Interactions between odor stimulus and test period for infant behavior.

Period	Olfactory stimuli	water	nf S	nf HM	cow M	nf FM	van	f M
Stimulus	nf AG	*0.002*	*0.014*	*0.014*	*0.000*	*0.001*	*0.000*	*0.008*
	water		ns	ns	ns	ns	ns	ns
	nf S			ns	ns	ns	0.055	ns
	nf HM				ns	ns	0.056	ns
	cow M					ns	ns	ns
	nf FM						ns	ns
	van							0.089
Post-stimulus	nf AG	*0.004*	ns	0.091	ns	0.068ns	ns	ns
	water		*0.004*	ns	*0.001*	ns	0.094	ns
	nf S			0.090	ns	0.067	ns	ns
	nf HM				*0.031*	ns	ns	ns
	cow M					*0.02*2	0.093	0.070
	nf FM						ns	ns
	van							ns

Matrix of p values of Fischer's LSD tests.

(Abbreviations: AG: secretion of areolar glands; S: sebum; HM: human milk; cow M: cow milk; FM: formula milk; van: vanillin; M: milk; f: familiar, nf: non familiar).

The ANOVA yielded a significant main effect of the stimulus on the duration of the newborns' oro-cephalic responses [*F*(7, 119) = 2.49; *p* = .02]. The duration of oro-cephalic actions when infants smelled the AG secretion was nearly double that when they inhaled any of the other odorants (Fischer's LSD tests, *p<.05* in all cases). Thus, AG odor elicited significantly longer responses than did the other homospecific substrates such as human milk and sebum, and than the heterospecific substrates such as cow's milk and cow's milk-based formula. Furthermore, the arbitrary odorant vanilla was poorly reactogenic as compared to the AG odor, ruling out that possibility that responses to AG secretion were caused by any odorant or by the effect of stimulus novelty.

The behavioral responsiveness elicited by AG odor also appears to be *temporally* distinguishable from that caused by the other stimuli. The infants exhibited greater reactivity to the AG secretion during its presentation than to any of the other odorants, as indicated by the marginally significant Stimulus by Test Period interaction [*F*(7, 119) = 2.04; *p* = .055; Figure 1 and Table 1]. Then, during the 10-sec following the stimulus period, oro-cephalic actions remained high in infants who were exposed to the AG odor. During this same post-stimulus period, the infants' responsiveness increased after having been exposed to sebum and cow milk odors (as compared to water, non-familiar formula milk and non-familiar human milk; *p<*.01). Thus, the behavioral impact of the AG odor appears to be both immediate and relatively long lasting in comparison with the impact of the other stimuli investigated here.

Finally, the AG secretion's odor from an unrelated lactating mother was followed by longer oro-cephalic responses than the milk (either natural or formula) that sated the infants during the first 3 postnatal days. Overall, given that we did not find a main effect of Mode of Feeding [*F*(1, 17) = .22; *p*>.05], nor any other interactions between stimuli, test period and mode of feeding, this suggests that the effects of AG secretion on neonatal behavioral activity does not appear to be strongly dependent on direct exposure to the secretion prior to the test.

### 2. Autonomic responsiveness

#### Maximum change of inspiratory amplitude (IA_max_)

**Figure 2:**
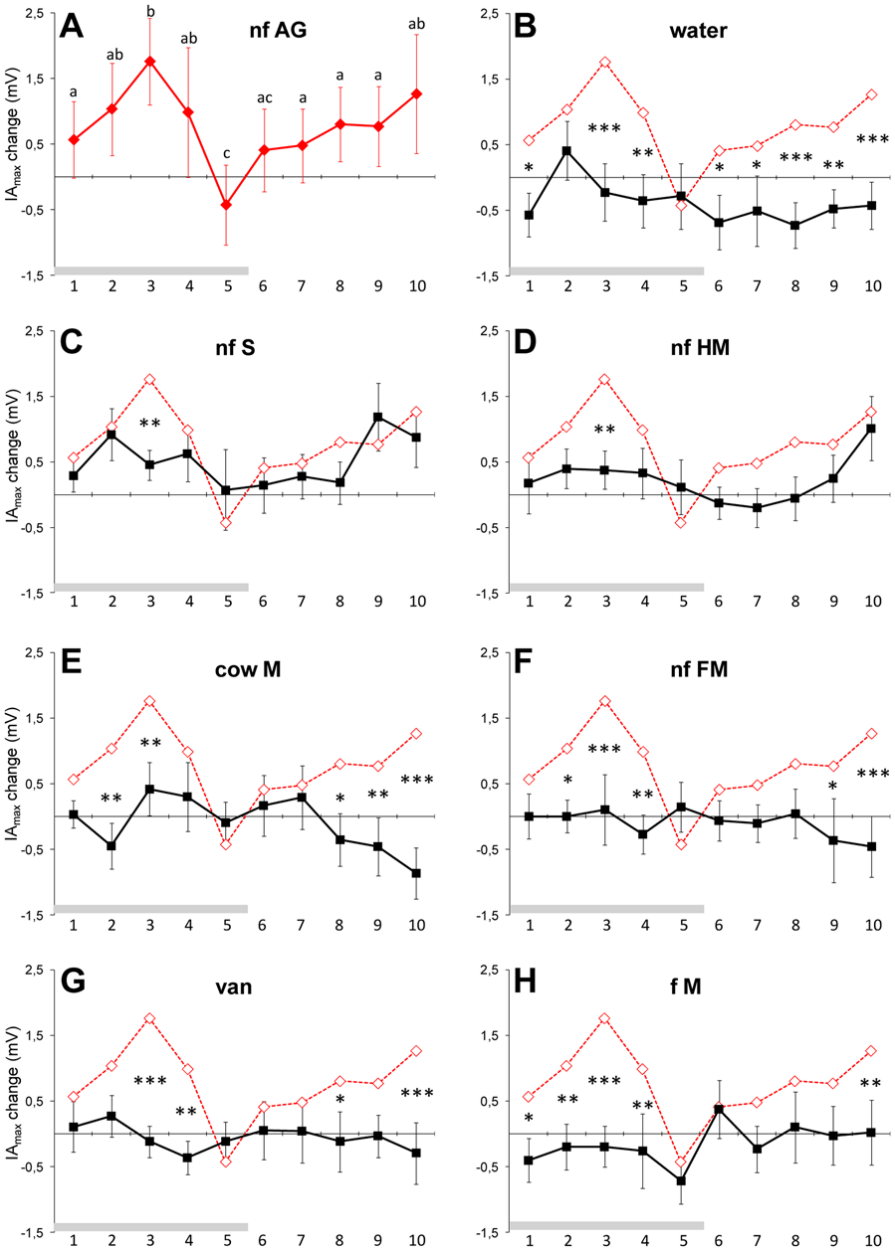
Inspiratory responses to areolar odor and to seven reference stimuli. Mean (± sem) of maximum change of inspiratory amplitude (IA_max_) in newborns during (2-sec stimulus blocks 1–5) and after (2-sec post-stimulus blocks 6–10) binarinal presentation of the following stimuli: A) areolar secretion; B) blank (water); C) sebum; D) human milk; E) cow milk; F) formula milk; G) vanillin; and H) habitual milk (mother's milk in breast-fed and formula milk in bottle-fed infants). Key to abbreviations in legend of Figure 1; values of IA_max_ in response to AG odor that differ significantly from the baseline are indicated by different letters; comparisons between the different stimuli (bold curves) and AG odor (red curves) are indicated by *, ** and ***: p<.05, .01 and .005, respectively; n = 16).

A main effect of the stimulation period was evidenced in the newborns' IA_max_ [ANCOVA, *F*(9, 117) = 2.81; *p<*.01]. This respiratory variable was significantly affected by the stimuli during the early (blocks 2–3) as compared with the later stimulus periods (blocks 5 and 8; *p*<.05). This stimulation effect is most marked for the AG odor [Stimulus × Test Period interaction: *F*(63, 819) = 2.93; *p*<.001], which appears to be the only stimulus that releases an immediate increase in IA_max_ among the set of stimuli administered that elicited an immediate increase in IA_max_ (Figure 2A).

As compared to the blank (water) stimulus, the variation in IA_max_ to AG secretion's odor confirms that this stimulus is clearly detected during stimulus blocks 1, 3, 4 and elicits sustained response for 10 sec. after its presentation is interrupted (Figure 2B). Further, the AG stimulus elicited higher IA_max_ than all other substrates from lactating females (especially in stimulus block 3; Figure 2C–D). The value of IA_max_ was also significantly higher in response to AG odor than to cow's milk and formula, respectively, (Figure 2E–F) during stimulus blocks 2, 3, 8–10, and 2–4, 9–10, for) and to vanilla during stimulus blocks 3, 4, 8, 10 (Figure 2G).

Finally, the odor of the milk used to feed the infants since birth was less active on IA_max_ than the AG odor (during stimulus blocks 1–4, 10; Figure 2H). No further main effect of the stimulus [*F*(7, 91) = 1.10; *p>*.05], mode of feeding [*F*(1, 13) = .05; *p>*.05] were found, nor were any interaction effects between these factors, were found. This supports the notion that human newborns' inspiratory responses to AG odor do not depend on previous exposure to a lactating breast.

#### Respiratory rate (RR) change

**Figure 3:**
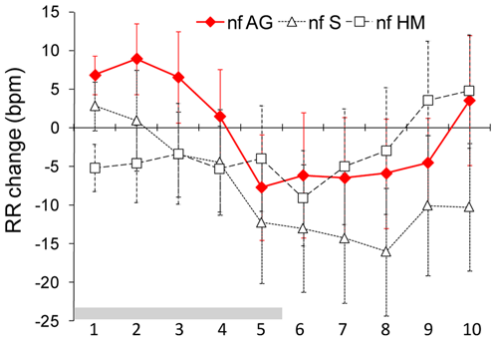
Respiratory rate in response to the odor of areolar secretion, sebum and milk. Mean (± sem) respiratory rate (RR) change of newborns during (2-sec stimulus blocks 1–5) and after (2-sec post-stimulus blocks 6–10) of the presentation of areolar secretions, sebum and human milk (key to abbreviations in legend of Figure 1; n = 16).

No significant main or interaction effects of odor stimulus, test period or mode of feeding were detected on RR change [*F*(7, 91) =  = .70; *F*(9, 117) = .64; *F*(1, 13) = .11, respectively; *p*>.10 in all cases]. Nevertheless, Figure 3 shows a higher RR change to the AG odor. The simultaneity of this non significant RR change with the significant increase in IA_max_ to AG odor suggests that this stimulus activates the newborns' respiration more than the other human substrates (milk and sebum). Again, this effect occurs independently from the newborns' previous exposure to breastfeeding.

#### Heart Rate (HR) change

**Figure 4:**
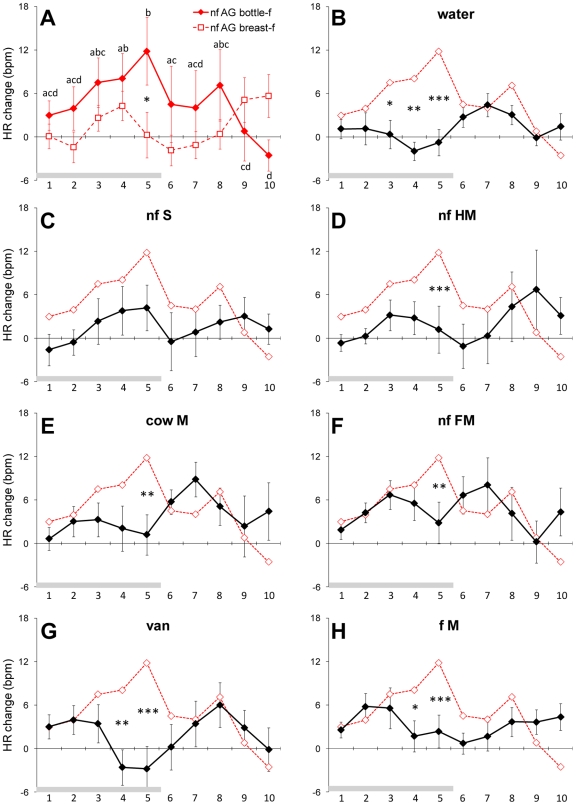
Cardiac response to the areolar odor and the reference stimuli. A) Mean (± sem) heart rate (HR) change of bottle- and breast-fed newborns in response to areolar secretions (n = 8 and 8; values of HR change of bottle-fed newborns in response to AG odor that differ significantly from the baseline are indicated by different letters); B to H) Mean (± sem) HR change of bottle-fed newborns during (blocks 1–5) and after (blocks 6–10) exposure to water (B), sebum (C), human milk (D), cow milk (E), formula milk (F), vanillin (G); and the infant's familiar milk (H) (Key to abbreviations, cf. Figure 1). Comparisons between the different stimuli (bold curves) and AG odor (red curves) are indicated by *, ** and ***: p<.05, .01 and .005, respectively; n = 8).

We found no significant main effects of the stimulus, test period and mode of feeding for this variable [*F*(7, 91) = .87; *F*(9, 117) = .91; *F*(1, 13) = .21, respectively; *p*>.10 in all cases]. We did, however, find a marginally significant 3-way interaction between stimulus, test period, and mode of feeding [*F*(63, 819) = 1.32; p = .051], which was due to a Period by Mode of Feeding interaction effect in HR response to AG odor only [2-way repeated-measures ANCOVA; *F*(9, 117) = 2.94; *p*<.005]. Post-hoc comparisons indicated that bottle-feeders, but not breast-feeders, responded by a regular HR increase during AG odor presentation (Figure 4A). This accelerative response of bottle-feeders reached significance on stimulus blocks 1–2 and 5, and ceased at stimulus withdrawal (Figure 4A). The AG odor-related HR acceleration differed from that observed to the presentation of the control stimulus (on blocks 3–5; Figure 4B), the homospecific substrates (on block 5; but not of sebum; Figure 4C–D), all heterospecific milks (on blocks 5 and 10; Figures 4E–F), vanillin (on blocks 4–5; Figure 4G), and the satiety-reinforced milks (on stimulus blocks 4–5 and 10; Figure 4H).

In contrast to the bottle-feeders, breast-feeders evinced a non-significant HR variation from baseline situated within a range of −1.81 and 8.08 beats per minute (bpm) (Figure 4A), a level of variation that is not different from these elicited by all other stimuli (p>.10 in all cases). This HR change difference between both groups of newborns may be caused by disparities in odor-induced motor responses. The newborns' HR change to AG odor is indeed positively linked with the relative duration of their oro-cephalic response to that stimulus (Pearson's r = .61; p<.05) and bottle-feeders tend to mouth longer to AG odor than do breast-feeders (mean ± SD: .304±.222 vs. .155±.136 during stimulus and .211±.284 vs. .119±.140, during post-stimulus).

## Discussion

The present research investigated whether 3 days-old human newborns can olfactorily differentiate the nascent secretion from the areolar (*viz.*, Montgomery's) glands of lactating females from other stimuli of human, other mammalian, or artificial origin. Results showed that newborns increased their behavioral and autonomic responses to the areolar secretion, indicating that they can detect them, and that they are attracted more to them than to the other stimuli. These findings provide the first direct evidence that newborns possess a selective and species-specific sensitivity to the secretion from Montgomery's glands. These points are discussed below.

### Detection of AG odor

The infants clearly sensed the AG odor, as they engaged significant oro-cephalic activation to it as compared to both odorless controls (i.e., against the pre-stimulus baseline and the blank water stimulus). Their reaction to AG odor was evident in a clear increase in duration of head and mouthing actions that, in line with prior work on human neonates [4], [17]–[20], are interpretable as reflecting interest and appetence.

The positive behavioral responsiveness to AG odor corresponded with an increase in the amplitude of inspiration and stable respiratory rate, a pattern of respiratory change indicating that the infants were stimulated to inhale the AG odor. Similar patterns associating enhanced respiratory amplitude and stable/decreasing respiratory rate have been described in adults in response to species-specific odorants and related to the activation of brain structures mediating olfaction [21]–[22].

Finally, the infants' HR response to AG odor was differentiated by the mode of feeding: whereas bottle-fed infants (i.e., those who were never exposed to the breast) evinced an overall accelerative response, breast-feeders showed an initial slight decelerative response followed by an acceleration. Cardiac deceleration is considered to be a reliable component of the orienting response in newborns exposed to tactile [23] or auditory stimulations [24], [25] in the absence of overt motor responses. In contrast, HR response is an acceleration in the presence of movement and is due to somato-cardiac reflex [23], [26]. Thus, the overall accelerative HR response found here most likely reflects the metabolic demand linked with augmented oro-cephalic actions triggered by the AG odor. The cardiac pattern of breast-feeders (deceleration then acceleration) may be explained by the concurrent effects of the somato-cardiac reflex (accelerative) and of the orienting response (decelerative). The somewhat lower motor activation to AG odor in breast-fed infants is probably due to the fact that the somato-cardiac reflex may not have completely swamped the decelerative effect of the orienting response.

In sum, while the interpretation of HR reactivity is more complicated, the other response measures suggest that 3-day-olds detect the volatile fraction released from human AG secretion. This finding is especially interesting because such a stimulus is virtually undetectable to adult humans (see Methods). It may be noted that such an extremely weak odor stimulus can reliably elicit autonomic and motor responses in sleeping infants when in contrast strong odorants do not alter adults' sleep states [27], [28]. In the early stages of ontogeny, the sleeping brain may thus remain sentient of an organism's odor environment. One tentative explanation of this pattern of findings would be that the detection of odorant volatiles from AG secretion firstly increased automatic attentional processes, as evidenced by the intensification of inspiration, and thus induced affective or motivational processing, as reflected in the stimulus-dependent increase of head-turning and mouthing actions.

### Response selectivity

The newborns responded to the AG odor in a selective way. First, they did not react to AG because it was the most intense (adult judges rated it very low in intensity; cf. Methods) or the least intense stimulus in the stimulus series (milk and sebum were given equally low intensity ratings). Second, the sensory activity of AG secretion cannot be attributed to a novelty effect as the most novel stimulus in the series, vanilla, was only weakly effective. Finally, and most importantly, AG odor was clearly differentiated in terms of both behavior and respiration from all other stimuli of homo- and heterospecific origin. This differentiation was actualized in response magnitude and temporal pattern. While AG odor elicited immediate motor and respiratory responses of high amplitude during the stimulus period, all other stimuli released lower magnitude responses that were slower to appear. A point worthy of mention here is that AG odor had a stronger appetitive impact than its supposed components (milk and sebum). This is a common occurrence in mammalian chemical communication, where splitting complex biological mixtures into fractions or elements often reduces their behavioral activity [29].

### Species-specificity

Within the limited set of heterospecific stimuli used here, the human newborns' response to AG odor appears to be species-specific. The responsiveness to AG odor was indeed clearly of higher magnitude and immediacy than to the odors of fresh cow's milk and cow's milk-derived formulas. Further, given that the AG secretions used here were collected from women that were not the infants' own mothers, it may be inferred that the functional activity of AG odor reflects a general property of human AG odor, rather than individual-specific features.

### Unconditional character of responsiveness to AG odor

Finally, the biological activity of AG odor on infants was assessed for its dependence on prior postnatal exposure to it. The present data indicate that oro-cephalic and respiratory responses were elicited regardless of the method of feeding. Thus, the absence of direct exposure with AG odor during the 3 days prior to the test did not have a marked effect on neonatal responsiveness, leading to the conclusion that the biological activity of AG odor in human infants may be based on inductive processes that do not depend on the postnatal environment.

In this connection, it is interesting to note that both motor and respiratory responses to AG odor clearly surpassed the same responses elicited by the infant's familiar milk, i.e. the milk (human or formula) that was repeatedly associated with maternal contact, sucking and satiety. Although the reinforcing properties of this stimulus are impressive at birth, the current findings do not provide any clues regarding the developmental origins of its powerful valence for the newborn and obviously call for additional investigations. One possible way could be that intra-amniotic experience with similar compounds is inductive of the observed postnatal effect (e.g., cf. [19]). Otherwise, indirect factors that remain to be characterized might contribute to the embryonic development of an unconditional stimulus-response loop. Research in other mammalian species has shown that the nipple emits chemosignals that have the potency to unconditionally control the behavior of nursing-naïve newborns [30]–[32].

### Areolar odor: potential effects on the onset of attachment

The arousing properties of areolar odor stimuli may favor the alignment of the infants' head with the mother's breast and ease the ensuing latching and sucking performance. Therefore, these stimuli may function to initiate the chain of behavioral and physiological events that lead to optimize the engagement and reinforcement of early interactive processes leading to the progressive establishment of attachment. Notably, breast chemosignals activate oral activity on the nipple [15], [16] that releases a cascade of behavioral, neural, neuroendocrine and endocrine processes in the newborn and the mother [33]. On the infant's side, these odor stimuli can only speed up the intake of the colostrum that, in addition to hydration, energy and immunity, brings in bioactive compounds affecting neonatal arousal, behavior and learning (e.g., prolactin, oxytocin [34]; opiate agonists [35], [36]; delta sleep-inducing peptide [37]; colostrinin [38]); otherwise, the areolar odor may be involved in the co-activation of other neonatal sensory systems involved in the development of perception of, and selective response to, the mother: for example, breast odor stimulates eye opening in infants [17], favoring early exposure to the mother's face. On the mother's side, an open-eyed newborn is a strongly reinforcing stimulus that affects the establishment of positive maternal responsiveness [39]. Furthermore, neonatal sucking activity released by, among other stimuli, mammary odors has potential impacts on structural and functional brain reorganizations, especially in the oxytocinergic neural networks of the hypothalamus (supraoptic and paraventricular nuclei) [40]. Specifically, the effects of sucking have been shown in various mammals and have been found to facilitate oxytocin release and nursing, as well as various aspects of maternal bonding [41], [42].

### Conclusion

This study demonstrates for the first time that exocrine glands situated on the areolae of lactating women emit volatile compounds that can reliably activate behavioral and autonomic responsiveness in human newborns. This extremely minute, most often overlooked, secretion is made up of the blended outputs from the sebaceous and lacteal structures that compose Montgomery's glands [15]. These areolar structures reach their functional climax during pregnancy and lactation and may accordingly have signaling, directional, and motivational roles for newborn infants facing their mother's breast for the first contacts or nursing episodes. Human infants' efforts to localize and orally seize a nipple are indeed not to be taken for granted, as a substantial proportion of infants exhibit initial difficulties in attaining this goal [43]–[45]. Our findings suggest that part of these adaptive difficulties may stem from practices that separate mothers and infants [46]–[48] and/or that consist of wiping off or masking areolar secretions and thereby reducing the probability of speeding up the highly protective first intake of colostrum [46].

Ongoing work examines how waking infants respond to the AG secretion in terms of both directional cuing and motivational bootstrapping. It appears especially important to investigate individual differences in the rate of areolar secretion by mothers and in the responsiveness to them by infants, as well as to correlate these differences with various events that are causal and consequential of the onset of attachment and bonding (endocrine factors in infant and mother, breastfeeding performance, infant thriving, and mutual mother-infant recognition). Finally, future work should chemically analyze this secretion to assess the behavioral activity of its components and to evaluate whether the whole mixture's activity relies on a limited set of volatiles (as seen in other mammals [30]) or on a complex mosaic of compounds.

## Methods

### Participants

This experiment was approved by the direction of the maternity of Dijon University Hospital, by the ethical committee of the CNRS, by Agence Française de Sécurité Sanitaire des Produits de Santé, and by the Committee for the Protection of Persons submitted to experimentation. Prior to entry of their infants into the study, the parents were informed about its aims and methods. They all gave written consent to let their infants participate, and were physically present during the experiment.

Twenty-two infants participated in the experiment. Data from 3 of them were dropped from the analyses because of unstable behavioral states during the tests. The mean age of the 19 remaining newborns (10 males, 9 females) was 74 hr at the test (SD = 7.1 hr; range = 64–85.5 hr). The infants' parents were of European origin and the mothers' age ranged from 23 to 38 years (mean ± SD = 29.9±3.9 years). They all had normal pregnancies (gestation duration: 39.1±8.1 weeks). The infants were in optimal health at birth (Apgar score ≥8 at 1 min and = 10 at 5 and 10 min; birth weight: 3459±418 g). Eleven were exclusively breastfed and 8 were bottle-fed.

### Behavioral States of neonates

Odor-elicited responses were recorded during periods of irregular sleep, as previous studies found that newborns display higher behavioral and autonomic reactivity to odors during this state [4]. The assessment of the infants' behavioral states followed Prechtl's definitional criteria [49]. The recording of both respiratory rate and behavior were used to determine the infants' states. As mentioned above, only 19 participants displayed behavioral signs of irregular sleep before feeding (3 infants had to be dropped due to either regular sleep or changing behavioral state during the test).

### Odor stimuli

Three types of stimuli were used: a) biologic mixtures of human or heterospecific origin, b) biologic mixtures reconfigured by industrial processes, and c) a pure odorant. This set of stimuli was selected to evaluate whether the behavioral effect of the AG secretion is differentiated from other species-specific/non specific odor stimuli, and how unfamiliar AG secretions match the effects of stimuli that are familiar to the infants.

The biological substrates consisted of: 1) non familiar secretion from AG, 2) non-familiar human milk, 3) non-familiar sebum, 4) familiar human milk, and 5) non-pasteurized cow milk (purchased at Ligny farm, Melun, France; sanitary license n° 7033801). Familiar substrates were obtained from the subjects' own mothers, while non familiar ones came from unrelated women matched for postpartum age with the mother. AG secretions were collected from 16 lactating mothers, and human milk and sebum came from 22 mothers. The infants were always exposed to the substrates from different, non familiar women.

AG secretions were taken whenever a non smoking breastfeeding woman undergoing an eventless postpartum noticed secretory AG. Before taking any sample, informed consent was obtained from donating women. The Montgomerian fluid was directly pipetted from an AG giving off visible secretions, introduced into an Eppendorf vial to be snap-frozen in dry ice. These samples were stored at −80°C until their use in a test. Right before the test, a fraction of AG secretion was thawed. Breast milk and sebum samples were collected within 5 min before a test. Assuming that the composition of sebum is qualitatively homogeneous across the epidermal surface [50], [51], the latter substrate was taken from women's forehead, a region bearing high sebum excretion rate [52]. On the evening prior to the test day, donor women were asked to cleanse their forehead skin with an alcoholic pad and instructed not to apply any cosmetics until sebum was sampled by rubbing a cotton pad on their forehead for 10 sec 5 min before the test.

The synthetic milks were made of formulas used locally [Modilac® and Nidal®, Nestlé, Vevey, Switzerland (n = 4 for each brand)] and of a non-familiar formula (Blédilait®, Blédina, Villefranche-sur-Saône, France). The odorous and blank control stimuli consisted in vanillin (Aldrich, Saint-Quentin-Favallier, France; concentration: 0.01% in distilled water) and in distilled water, respectively.

Because the olfactory responses of human newborns are positively correlated with stimulus intensity [53], we controlled for differential effects of stimulus intensity. Twelve adult subjects (mean age ± SD = 28.2±5.4 years; 6 females) rated the subjective intensity of the stimuli on a 9-point Likert scale [range: 1 (not at all intense) to 9 (extremely intense)]. A repeated-measures ANOVA yielded a main effect of stimulus on intensity ratings [F(7, 77) = 19.97, p<.0001]. All odorants of non-human origin were rated as more intense (familiar formula milk: M ± SD = 2.42±.67; non-familiar formula milk: 2.33±.65; cow milk: 2.08±.79; vanillin: 1.67±.65) than the odorants of human origin (non-familiar AG: 1±.21; non-familiar sebum: 1±.25; non familiar human milk: 1±.18) (Tukey tests, *ps*<.05). The latter odorants were rated similarly and without difference with the blank (water: 1±.21).

### Procedure and Recording Material

For testing, the newborns were sat in a semi-reclining chair in a quiet room in which thermal, sound, and lighting ambiance was held as constant as possible. Light was set at a dim level and the room temperature was between 23–27°C. The infants were tested on average 130±47 min. after the last feed. Prior to testing, two experimenters placed the biosensors on the infant's arms (heart rate) and abdomen (respiration). The physiological parameters were continuously recorded using an 8-channel MacLab data-recording system (ADInstruments Pty Ltd, Castle Hill, Australia) that was connected to a laptop computer. The electrocardiogram was recorded using repositionable pre-gelled pediatric electrodes (BB-COM 2, Comepa, Saint-Denis, France) placed just above the wrists on the insides of the right and left arms (Lead I). An electronic filter was used to attenuate unwanted frequency components of signals linked to movement artifacts (low pass-filtering at 50 Hz). Abdominal breathing was recorded from a pneumobelt (Model 1132, Pneumotrace, UFI Instruments, Morro Bay, CA) strapped around the infant's abdomen. Heart (HR; in bpm) and respiratory rates (RR; in bpm) were recorded online and the maximum amplitude of inspiration (IA_max,_ in mV) was calculated off-line using the Chart software (version 3.5.2.). HR change is a relatively accurate index of metabolic demand and may reveal processes linked to attention and orienting response [54], [55], whereas odor-elicited changes in respiratory activity have been shown to be a reliable index of stimulus detection in sleeping newborns [4], [20], [56]. Distinct respiratory parameters (i.e., RR and IA_max_ or depth of breathing) were recorded because previous studies in adults demonstrated that they are dissociable and differentially correlated with cerebral responses [21], [22]. The mouthing and cephalic movements of newborns were recorded throughout the session with a silent digital video camera focused on a frontal view of the infant's face.

The testing was run by 3 experimenters. Experimenter 1, who was blind to the nature of the odorants, stood behind the infant to administer the stimuli prepared right before the test by Experimenter 2. The 8 stimuli were administered in a random order for each infant. To avoid any unwanted odor emanating from the presenting Experimenter's hand, any use of odorous soap was avoided and 20 cm-long glass sticks were used. The tip of the stick carrying each stimulus was posited binarinally at 0.5–1 cm under the nostrils. The onset and offset of each 10-sec stimulus trial were entered on the polygraphic recordings by Experimenter 3 (also blind to the nature of the stimuli) who followed the infants' behavior through a TV monitor and the psychophysiological recordings, and who controlled stimulus duration and inter-stimuli intervals (minimum: 50 sec).

### Autonomic responses

For each odor trial, the dependent variables were computed off-line from HR, RR and IA_max_, using the Chart application software, by subtracting the mean of data during the 2 sec pre-stimulus block (baseline condition) from the mean of data during the subsequent 2 sec-blocks of the stimulus period and of the post-stimulus period. The physiological data from 3 participants were excluded due to technical problems. Thus, the statistical analyses on autonomic responses involved 16 newborns (8 males/8 females; 8 breast-/8 bottle-feeders).

### Behavioral responses

The newborns' videotaped oro-cephalic responses were coded to provide indices of attraction and appetitive pre-ingestive responses. They were conservatively coded as exclusive items (cephalic actions that occurred with mouthing were not scored) using Adobe Premium Pro software (Adobe Systems Inc., San Jose, California, USA). The following mouthing actions were taken into account: rooting, munching, tongue or lips protrusions, licking, and sucking (see definitions in [17]; cf. Figures 1B–C). The cephalic actions were any slight head movement while the nose was above the stimulus. The selected items were viewed in slow motion and frame-by-frame to time their onset and offset, with a precision of ±1 video frame (i.e., ±.04 s). For each stimulus, the duration of mouthing and head movements was computed by subtracting the mean value during the 10-sec prestimulus block (baseline condition) from the mean of the data during the subsequent 10-sec blocks of the stimulus and of the post-stimulus periods. The behavioral variable submitted to statistical analyses was calculated by pooling the relative durations of mouthing and head movements (total duration of behavioral items/10 sec) to provide an index of oro-cephalic activation by the olfactory stimuli.

Inter-observer reliability was assessed by a second coder who was blind to the nature of the stimuli. He viewed independently a sample of 30 video clips of the infants' mouthing and cephalic movements. Spearman correlation coefficients computed on the duration of mouthing and cephalic movements among the two coders were 0.88 and 0.91, respectively.

### Statistical Analyses

Preliminary analyses verified whether the autonomic and behavioral measures were related to the time elapsed since the beginning of the last newborn's feed. It appeared that HR, RR, and AI_max_ following certain stimuli (i.e., areolar gland secretion, human milk, sebum, formula milk, water) were significantly correlated with the time elapsed since the last feed, in line with previous investigation [20]. Accordingly, the time elapsed since the newborn's last feed was entered as a covariate using an 8×10×2 (olfactory stimuli × test period × mode of feeding) analysis of covariance (ANCOVA), with the mode of feeding (breast vs. bottle) as between-subjects factor and odor stimulus and test periods as within-subjects factors.

For the oro-cephalic responsiveness, the effects of the independent variables (olfactory stimulus, test period and mode of feeding) were assessed using a 3-way repeated-measures ANOVA. Preliminary analyses for gender effects yielded no significant differences on both the autonomic and behavioral data making it possible for us to exclude the gender factor from further analyses. Fisher's least significant difference (LSD) test was used for the post hoc multiple comparisons between means.
